# Supplementary material for: Favorable Effects of Voriconazole Trough Concentrations Exceeding 1 μg/mL on Treatment Success and All-Cause Mortality: A Systematic Review and Meta-Analysis
Source: J Fungi (Basel). 2021 Apr 16;7(4):306. doi: 10.3390/jof7040306 (PMC8072959; doi:10.3390/jof7040306)
Supplement: Supplementary file 1 [file jof-07-00306-s001.zip › jof-1174708-supplementary.pdf]

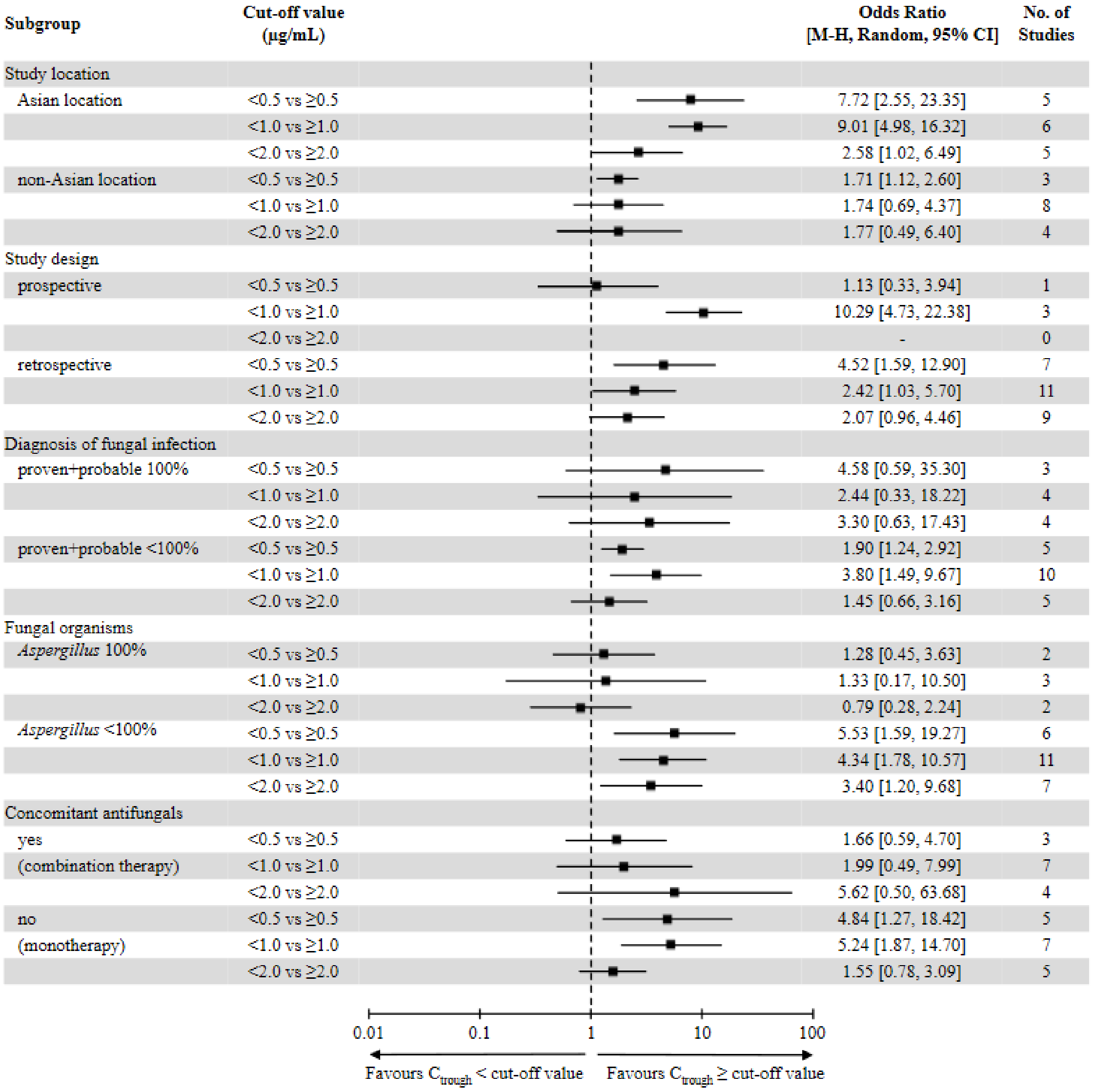

Figure S1. Summary of subgroup analyses for treatment success. Data are presented as forest plots of the odds ratios (ORs) for treatment success according to prespecified baseline subgroups. ORs and 95% confidence intervals (CIs) were calculated using the Mantel–Haenszel (M-H) method according to the random-effects (random) model.

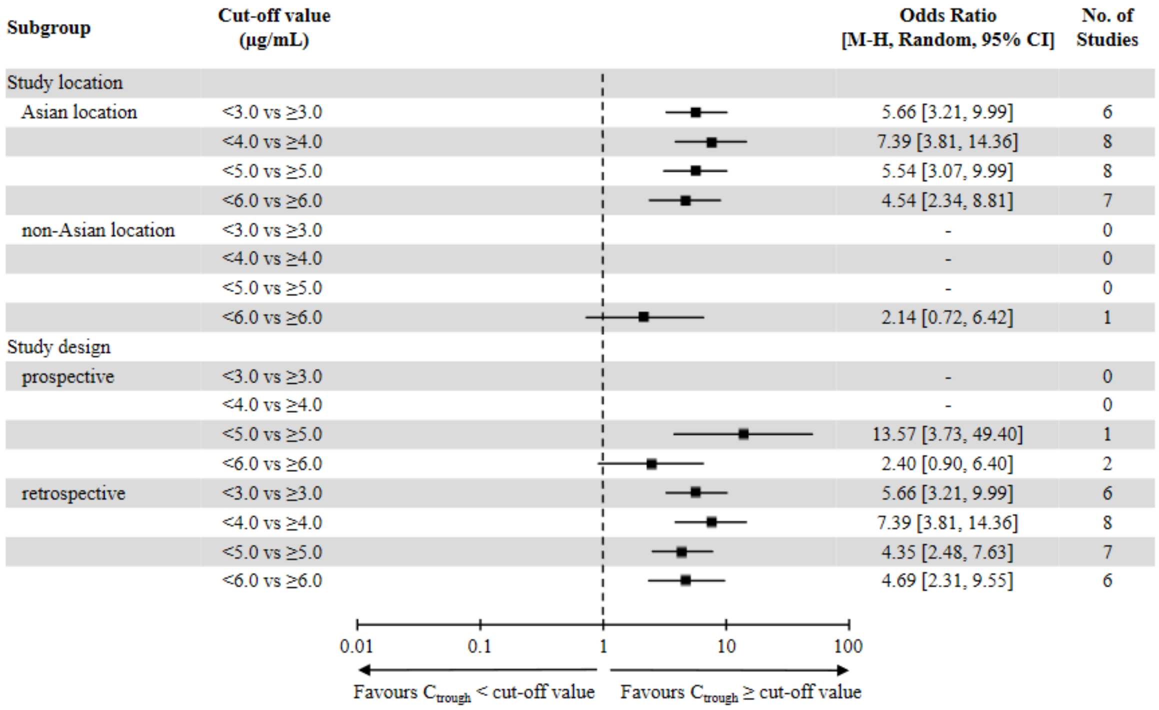

Figure S2. Summary of subgroup analyses for hepatotoxicity. Data are presented as forest plots of the odds ratios (ORs) for treatment success according to prespecified baseline subgroups. ORs and 95% confidence intervals (CIs) were calculated using the Mantel–Haenszel (M-H) method according to the random-effects (random) model.

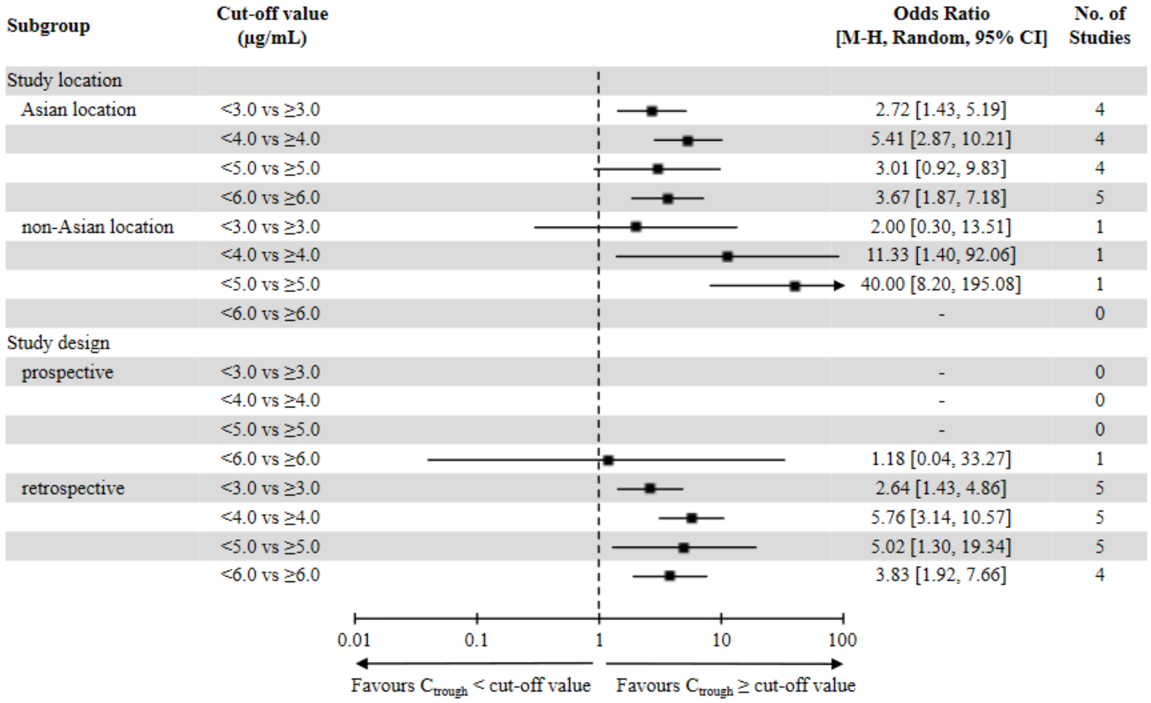

Figure S3. Summary of subgroup analyses for neurotoxicity. Data are presented as forest plots of the odds ratios (ORs) for treatment success according to prespecified baseline subgroups. ORs and 95% confidence intervals (CIs) were calculated using the Mantel–Haenszel (M-H) method according to the random-effects (random) model.
